# Governance Challenges of Smallholder Agricultural Carbon Projects and the Potential of Digital Tools: Insights from Kenya

**DOI:** 10.1007/s00267-026-02410-7

**Published:** 2026-02-17

**Authors:** Vida Mantey, Regina Birner, Athena Birkenberg, Christine Bosch, Viviane Guesbeogo Yameogo, John Mburu

**Affiliations:** 1 Hans-Ruthenberg-Institute of Agricultural Sciences in the Tropics, University of Hohenheim, Stuttgart, Germany; 2 Department of Agricultural Economics, University of Nairobi, Nairobi, Kenya

**Keywords:** Carbon certificates, Governance analysis, Payment for ecosystem services, East Africa, Voluntary carbon market, Bargaining power

## Abstract

Agricultural carbon projects are increasingly promoted as instruments to address climate change while supporting sustainable agriculture. These projects combine sustainable land management (SLM) practices with a carbon credit component, creating complex governance structures involving diverse actors with unequal power. Understanding the governance challenges that may impede their effective implementation is therefore essential. Despite growing evidence on the potential of digital tools in agriculture, their role in agricultural carbon projects remains underexplored. This study employs a qualitative case study of two pioneering carbon projects in Kenya, alongside a participatory and visual mapping tool (Process Net-Map), to engage with stakeholders. It combines concepts of principal-agent and bargaining power theory to analyse the governance challenges of agricultural carbon projects, and the potential role of digital tools in addressing them. The findings reveal layered principal-agent relationships in the carbon credit component, characterized by strict monitoring and external accountability, exacerbating information asymmetries and shifting performance risks to actors with limited bargaining power. While women play a pivotal role in implementation and monitoring, intra-household power relations constrain their control over assets and benefits, thereby reinforcing gender inequities. Digital tools currently support data collection and reporting, with potential to reduce transaction costs and improve accountability, but their use remains largely confined to the SLM component. Expanding digital tools beyond monitoring to support participation, transparency, and feedback could strengthen smallholder bargaining power. The current study contributes to literature by highlighting how the carbon credit component reshapes and intensifies existing SLM governance challenges and offers insights for project developers and policymakers seeking to promote more equitable and effective agricultural carbon initiatives.

## Introduction

The development of agricultural carbon projects is part of a wider effort to promote sustainable agriculture, address climate change and meet global greenhouse gas (GHG) emissions reduction targets. Agricultural carbon projects can be defined as agricultural land management projects with a carbon credit component. They offer the opportunity to finance the promotion and adoption of sustainable agricultural practices from which smallholder farmers benefit for their livelihoods. These practices not only reduce GHG emissions but also enhance farm productivity by improving soil fertility and increasing farmer resilience and adaptation to climate change (Hayo and Hasegawa 2024; Okyere and Kornher 2023). Smallholder Agricultural Carbon Projects (SACPs^1^) connect farmers to international voluntary carbon markets to receive funding in exchange for carbon sequestered on their farms (Hayo and Hasegawa 2024; Paul et al. 2023). Sustainable practices promoted by SACPs include Verra’s Sustainable Agricultural Land Management (SALM^2^) project type, which include minimum tillage, residue management, cover crops, intercropping, composting, agroforestry, and dairy management practices. SACPs mimic payment for ecosystem services (PES) schemes, in the sense that they reward for generated ecosystem services generated by landholders. However, finance is specifically tied to verified carbon sequestration and credit issuance (and not just compliance with practices), and therefore, includes a complex monitoring system, requirements set by international standards and is a market-based scheme struggling with price volatility and time-lags in finance. The need to bring together thousands of smallholder farmers in complex carbon schemes raises important governance challenges that may affect the success of their implementation.

While there are high expectations for SACPs to reduce GHG emissions and improve smallholder livelihoods (Gordon et al. 2021), there is little systematic evidence on the governance challenges affecting their implementation. Previous studies have focused on the effects of carbon projects on productivity and livelihoods (Nyberg et al. 2020), the role of carbon credits in incentivizing climate-smart farming (Hayo and Hasegawa 2024), the impact of carbon farming training on welfare (Okyere and Kornher 2023), approaches to quantifying soil organic carbon (Okoli and Birkenberg 2024; Guan et al. 2023), the challenges of engaging smallholders (Lee, 2017; Foster and Neufeldt 2014), and the role of intermediary organisations (Lee et al. 2016). In their analysis of pro-poor carbon market projects in East Africa, Lee et al. (2016) identified time lags between adoption and carbon revenue distribution, knowledge gaps about voluntary carbon markets, and insignificant carbon payments as major challenges in SACPs. Similarly, Cavanagh et al. (2021) reiterated the insignificance and low value of carbon payments in the Kenyan Agricultural Carbon Project, arguing that this could threaten participants’ adaptation and mitigation efforts. However, it is unclear why these challenges arise and how they relate to different components and stages of implementation. Empirical studies on the roles of different actors and the specificities of SACPs are scarce. Previous studies on Reducing Emissions from Deforestation and Forest Degradation (REDD + ) have identified equity issues in benefits distribution and emphasised the importance of focusing research on procedures and decision-making, and the overall context in which these projects occur (see, for example, Pascual et al. 2014; McDermott et al. 2013). However, forest carbon projects differ in scope and implementation, and it is more challenging to engage large numbers of smallholders, as is the case with SACPs.

Furthermore, despite a large body of literature on digital tools in agriculture (see, for example, Birner et al. 2021; Daum et al. 2019), there has been little discussion of how digital tools can improve institutional mechanisms. Previous studies have focused on approaches to quantify GHG emission reductions or soil organic carbon (e.g., Okoli and Birkenberg 2024; Guan et al. 2023), neglecting their role in the overall implementation process of SACPs. While Cavanagh et al. (2021) raised critical concerns about the need for research on the design and structure of SACPs, Schilling et al. (2023) echoed the need to understand the institutional framework of SACPs. Empirical studies that systematically assess the implementation of SACPs and the role of digital tools are lacking.

This paper therefore develops a systematic framework for analysing governance challenges in SACPs and applies this framework to identify underlying reasons for the observed challenges. It also discusses the role of digital tools in SACPs. Specifically, this study seeks to answer the following research questions:

- What are the governance challenges inherent in the implementation of smallholder agricultural carbon projects, their underlying reasons, and mitigation strategies?
- What is the role of digital tools in supporting successful (more inclusive) carbon projects?

To answer these questions, a qualitative case study was conducted on two SACPs in Kenya: the Livelihoods Mt. Elgon Project (Case 1) and the Kenya Agricultural Carbon Project (Case 2). These cases are interesting because Case 2 is the first soil and agricultural carbon finance project in Africa to implement SALM (Tennigkeit et al. 2013), and Case 1 is the first to incorporate a dairy component. Furthermore, Kenya’s numerous information and communication technology start-ups provide a backdrop for increasing digitalization (Stroisch, 2018). These projects differ in terms of their scope and implementation mechanisms. To better understand the governance challenges in the SACPs, principal-agent (P-A) theory and bargaining power theory were applied. Additionally, a participatory mapping tool–the Process Net-Map—was used to map actors, their roles and linkages, levels of influence, and governance challenges in the SACPs implementation process, following Birner and Sekher (2018). Details on the application of the Process Net-Map are presented in Section “Description of the Process Net-Map tool ”.

## Conceptual Framework

This section begins by describing the structure of SACPs and identifying the key actors and their respective roles. It then sets out the theoretical principles that underpin the analysis, paying particular attention to principal–agent relationships and bargaining power dynamics. Finally, it examines benefit-sharing arrangements within SACPs, situating them within these theoretical frameworks to demonstrate the influence of project design on the distribution of benefits.

### Structure of SACPs

**Fig. 1:**
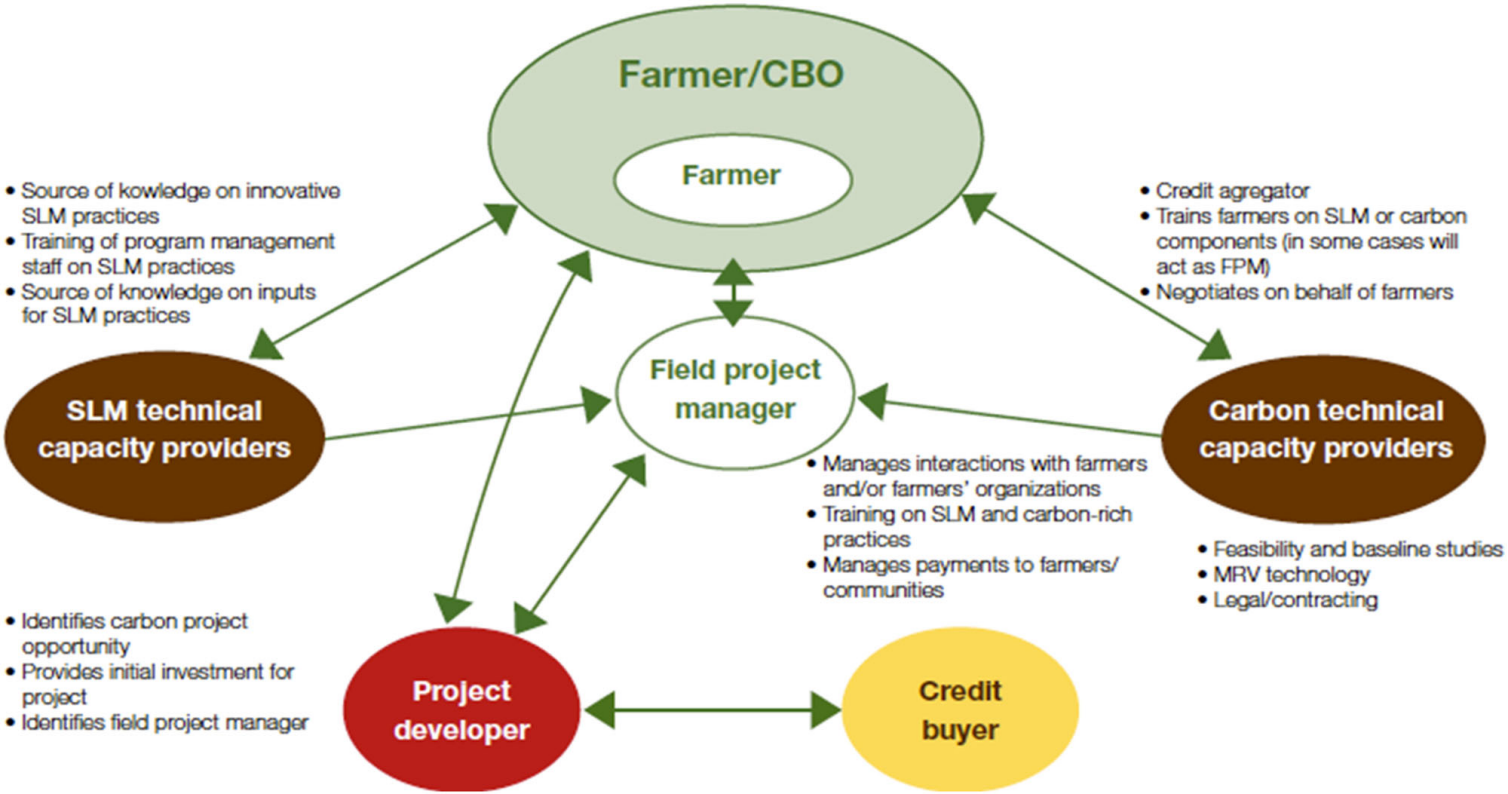
Key roles and functions in a generic model of smallholder agricultural carbon projects (Shames et al. 2012)

The generic structure of an SACP, as depicted by Shames et al. (2012) is shown in Fig. 1. It is a central proposition of this paper that, for analytical purposes, an agricultural carbon project can conceptually be divided into two components, referred to here as the “SLM component” and the “carbon credit component”. The SLM (Sustainable Land Management) component is largely equivalent to any conventional development project that aims to encourage farmers to adopt SLM practices. Such projects have been promoted for decades by international development organizations and national governments with the aim of making agricultural production more environmentally sustainable and protecting natural resources, such as soil. SLM projects typically involve actors depicted in the center and on the left-hand side of Fig. 1. They include a project developer, which could be a development organization such as the World Bank or a bilateral agency. They engage a field project manager such as a government agency or a non-governmental organization. This organization uses extension agents, referred to as “SLM technical capacity providers” in Fig. 1. They train farmers, who are typically organized in farmer groups (referred to as community-based organizations (CBOs) in Fig. 1).

The opportunities and challenges of SLM projects are well documented in the literature (Pan et al. 2022; Kim et al. 2021; Foster et al. 2017). While the environmental benefits are evident, the economic benefits are not, therefore encouraging farmers to adopt SLM practices has proven to be challenging. This is because SLM practices are often labor-intensive, and the benefits are not immediately visible, but rather occur in the long run (Foster et al. 2017; Lee, 2017). Moreover, like other agricultural development projects, SLM projects are confronted with the typical problems involved in the provision of agricultural advisory services (Aremu and Reynolds 2024; Anderson and Feder 2004), which include a lack of incentives for extension staff due to information asymmetry and resulting P-A problems (detailed in Section “Structure of SACPs”). It is inherently costly for an agency that hires extension agents to supervise them, as their activities are spatially dispersed. Moreover, the results of the extension activities, in this case the adoption of SLM practices, partly depend on factors beyond the control of extension agents (Birner et al. 2009). Extension services often suffer from insufficient financial resources since extension has the characteristics of a public good and a merit good, resulting in a low willingness of farmers to pay for it (Anderson and Feder 2004).

SACPs add a “carbon credit component” to such SLM projects. This requires the inclusion of two additional types of organizations, as shown in Fig. 1: “Carbon technical capacity providers” and “carbon buyers.” The promise of SACPs is their potential to resolve some of the long-standing problems of SLM projects by generating financial resources. These resources can be used (1) to better finance the SLM technical capacity providers, and (2) if the carbon revenues are shared with farmers, to create incentives for them to adopt SLM practices. Moreover, the monitoring activities conducted in the carbon component can also improve the monitoring of the SLM component. This is because the carbon credit component requires the project to provide evidence that the SLM technical capacity providers were successful, i.e., that the SLM activities were adopted by the farmers. By adding this monitoring activity, the carbon credit component of an agricultural carbon project can reduce the information asymmetry and the P-A problems that exist for P-A relations, as discussed in Section “Theoretical considerations”. Phrased differently, the carbon credit component adds additional accountability to an SLM project.

The inclusion of a carbon credit component into an SLM project, however, presents its own set of challenges. Information asymmetry and a resulting P-A problem exist between the carbon technical capacity providers and the field project manager, as well as between the project developer and the carbon credit buyers. Certification and verification systems, which are implemented by the carbon technical capacity providers, aim to address this challenge.

If one conceptualizes an agricultural carbon project as sketched above, two interesting questions arise from a governance perspective:

- How should SACPs be organized to harness the potential synergies between the SLM component and the carbon credit component to reduce the governance challenges arising from information asymmetries in either component of the project?
- How should the financial revenues from carbon credits be distributed among all relevant organizations to create sufficient financial incentives to address typical problems of SLM projects (low adoption rates, insufficient resources for technical capacity development)?

To answer these questions, the literature that has analyzed the governance problems in SLM and agricultural carbon projects, as well as related literature on PES and REDD+ schemes is reviewed. To address the first question, this study focuses on articles that have applied an information-asymmetry / P-A framework, since—as the above description shows—information asymmetries are a major underlying reason for governance challenges in both the SLM and the carbon components of SACPs. To address the second question, the literature that has addressed questions of benefit sharing in agricultural carbon projects and PES and REDD+ schemes is reviewed.

### Theoretical Considerations

The study combines concepts of principal-agent and bargaining power theory to examine the aforementioned dynamics in SACPs. This helps elucidate the governance challenges in SACPs that emanate from information and incentive issues, as well as power asymmetries that shape contracts designed to address them. This is consistent with empirical reviews which have noted that imperfect information and institutional power asymmetries hinder bargaining and contract negotiation in PES schemes (Milne and Chervier 2014). P-A theory examines relationships in which principals delegate tasks to agents whose actions are imperfectly observable and whose incentives may differ from those of the principal (Williamson, 2000; Arrow, 1985; Ross, 1973). In SACPs, delegation occurs through a chain of relationships involving project developers, technical service providers, field managers, farmers, and carbon buyers (see Table 1).

**Table 1:** Principal-agent relationships among SACP actors

Principal	Agent	Relation/explanation	Sources of bargaining power
***SLM component***			
Project developer	Field project manager	Project developer mandates field project manager to supervise project implementation and expects adoption of sustainable practices	Control over project funding, contracts, and strategic decisions
Field project manager	SLM technical capacity provider	Field project manager requires SLM technical capacity provider to provide extension services and SALM technical support to farmers and expects monitoring reports	Operational authority and coordination role
SLM technical capacity provider	Farmer/community-based organization (CBO)	SLM technical capacity provider requires farmers/CBO to support in training farmers on sustainable practices, data collection, and reportin,g and expects accurate reporting	Control over access to training, inputs, and project participation
***Carbon credit component***			
Project developer	Carbon technical capacity provider	Project developer mandates carbon capacity provider to train project field managers and SLM technical capacity providers on carbon monitoring, reporting, and verification technologies, quantify emission reduction, and expects accurate estimates and increased carbon credit	Control over contractual engagement and data ownership
Carbon technical capacity provider	Field project manager	Carbon technical capacity provider requires project field manager to provide monitoring data and expects accuracy	Technical expertise and control over MRV protocols
Carbon buyer	Project developer	Carbon buyer seeks certified carbon credit from project developer and expects accurate representation of the indicated emission reduction	Market access, price-setting power, certification demand

Source: Authors

In the SLM component, P-A issues mirror those typically encountered in agricultural development projects, encompassing moral hazard and adverse selection (Stiglitz, 1987; Arrow, 1985). For instance, inadequate incentives and minimal oversight can diminish the quality of extension services in SACPs (Mantey et al. 2026), while smallholders’ preference for immediate benefits may diverge from developers’ long-term carbon goals (Cavanagh et al. 2017). As actors often alternate between principal and agent roles, they may exploit informational advantages. This underlines the need for better incentive alignment, monitoring, and shared decision-making (Namyenya et al. 2023; Taylor, 2004).

The carbon credit component adds a more hierarchical governance layer. Project developers delegate carbon monitoring and accounting to specialized technical providers, while carbon buyers act as upstream principals seeking verified and certified emission reductions. This layer introduces stricter monitoring, third-party verification, and penalties for non-compliance. This reflects the commodification of emission reductions, and the need to manage credibility and reputational risk in carbon markets (Schneider and La Hoz Theuer 2019). It may result in shifting responsibilities for performance and risk to lower-level actors, as has been widely observed in value-chain and certification-based governance systems (Hambloch et al. 2026). Information asymmetries in largely unregulated voluntary carbon markets can facilitate greenwashing, and the high costs and technical complexity of monitoring and verification can encourage inaccurate or incomplete reporting (Tennigkeit et al. 2023; van Kooten, 2017). Although sanctions and verification mechanisms exist, these may be underused due to financial costs and the risk of eroding local trust. This can lead developers to tolerate some level of non-compliance (Ezzine-de-Blas et al. 2016). Effective governance requires incentives and oversight to align agents with project objectives (Schieg, 2008).

Although P-A theory supports the identification of information and incentive issues, it does not fully explain the observed outcomes of governance in SACPs. This paper therefore uses bargaining power theory to explore how control over resources, institutional authority, and outside options influence contractual terms and benefit distribution (Hambloch et al. 2026; Agarwal, 1997). Bargaining power in SACPs is highly asymmetrical: carbon buyers and project developers control access to finance, certification, data infrastructures, and markets, while smallholders have limited alternatives. These asymmetries constrain the set of feasible contracts and influence the allocation of risks, responsibilities, and benefits across the project chain, often to the disadvantage of local actors (Hambloch et al. 2026). Market power and incentive structures determine how effort is provided and benefits are distributed in PES (Schneider and La Hoz Theuer 2019). Gender norms further influence bargaining power in agrarian settings (Agrawal, 1997). At the intra-household level, women act as effort-providing agents while men retain residual claimant status (Agarwal, 1997; Sen, 1987).

### Benefit Sharing in Agricultural Carbon Projects

SACPs are not only designed for environmental outcomes but also aim to generate significant economic and social co-benefits (Hayo and Hasegawa 2024; Foster et al. 2017). Benefit sharing, which is defined as the deliberate distribution of monetary and/or non-monetary carbon benefits among stakeholders who contribute to emission reductions, is a core element of voluntary carbon markets (World Bank Group, 2019). However, empirical studies have questioned the effectiveness and fairness of these arrangements, highlighting high transaction costs, limited transparency in revenue allocation and generally low, irregular payments to farmers (Howard et al. 2016; Shames et al. 2016; Atela, 2012). These challenges are exacerbated by the complexity of SACPs, limited trust in project developers and concerns over project sustainability in the long term (Sipthorpe et al. 2022; Lee et al. 2016).

P-A theory is useful for analyzing governance challenges in benefit sharing, especially those arising from information asymmetry between project developers, intermediaries, and farmers or community-based organizations (van Kooten, 2017; Howard et al. 2016). Project developers, acting as principals, may be unable to adequately observe agents’ actions, creating risks of moral hazard and adverse selection, while agents may withhold information to further their own interests. These dynamics can affect the fair distribution of benefits and the selection of suitable implementing partners (Basak and van der Werf 2019). Addressing these issues requires participatory benefit-sharing mechanisms that incorporate local perspectives, align project objectives with farmers’ priorities and strengthen transparency, accountability, and trust among stakeholders (Tennigkeit et al. 2023; Lee, 2017; Howard et al. 2016).

## Research Design and Methodology

### Research Design

A qualitative case study design was employed to gain insight into the reasons for, and timing of, governance challenges arising in SACPs, and how these challenges can be mitigated. The two cases selected for this study differ in terms of their implementation methods and funding sources.

The first step of the study was a review of literature and documents from organisations involved in the projects, such as project developers, donors, and investors. This provided a better understanding of the actors involved in the projects and helped to triangulate and validate the findings (Adu-Baffour et al. 2021). The review provided information on the goals, objectives, mitigation practices, carbon accounting methodologies, and certification processes. The first author also attended a training session for field staff on digital tools for data collection, organised by ‘UNIQUE’, a carbon technical capacity provider. Stakeholders were then identified and purposefully selected based on their experience, knowledge, and involvement in the two cases, with the assistance of the project managers and using snowball sampling. Due to the potential sensitivity of the information, and to ensure the anonymity of the respondents, the institutional affiliations of the respondents are not reported. Stakeholders were selected from among project funders (donors and investors), project developers/implementers, government parastatals, CBOs, dairy cooperatives, farmers, and farmer groups involved in the projects. A total of 85 interviews were conducted, including 10 focus group discussions (FGDs), each involving 10-20 members. To reduce potential sampling bias, these discussions were held within 10 randomly selected farmer clusters drawn from 10 of the 15 project zones. The FGDs provided additional insights into participants’ perspectives and perceptions of the SACPs.

### Overview of the Case Study

The Livelihoods Mt. Elgon project (Case 1) and the Kenya Agricultural Carbon Project (Case 2) were implemented in Trans-Nzoia and Bungoma counties in Western Kenya by Vi Agroforestry (project developer), a Swedish NGO. The objective of both projects is to improve the livelihoods of smallholder farmers through improved food production from SALM and dairy management practices, while reducing greenhouse gas emissions. In addition to generating carbon credits, the project developers hope that the adoption of SALM, which includes agroforestry, residue management, cover cropping, reduced or zero tillage, manure management, and dairy farming (Atela, 2012), will improve soil fertility. Table 2 shows the characteristics of these projects.

**Table 2:** Characteristics of the two cases

Characteristics	Case 1	Case 2
Project implementation period	2016–2026	2009–2029
Project developers/implementers	Vi Agroforestry	Vi Agroforestry
Type of funding	Investor fund	Donor fund
Targeted farmers	15,000 smallholders including 70% dairy farmers	60,000 smallholder farmers
Carbon accounting standards	Gold standard: GHG reduction due to increased dairy productivity and Verra: SALM	Verra: only SALM
Expected sequestration during project implementation period	One million tons of carbon dioxide equivalent (CO₂e) on 25,000 ha	1.23 million tons of CO₂e on 45,000 ha
Farmer benefit	Extension service provision	Direct cash payment and extension service provision

### Data Collection and Analysis

Data were collected using FGDs and key informant interviews between May and September 2022. A participatory and visual mapping tool called Process Net-Map was used. Process Net-Map allows the sequential steps of a process to be mapped (Birner and Sekher 2018). The tool provides insights into the different stages of SACPs, offering the advantage of visualisation and the identification of actors’ power or influence on the outcome (adoption of SALM and dairy practices). Process Net-Map is designed to provide insights into governance challenges in the implementation of complex programs and projects and to elicit implicit knowledge about such projects (Birner et al. 2011; Raabe et al. 2010; Schiffer 2007). The tool was used in this study to assess all stages involved in SACPs and to identify the actors and governance challenges associated with each stage. To ensure a spectrum of stakeholders, we made sure that all relevant actors were represented. Separate interviews were conducted with women. Data collection in Case 2 was limited because the monitoring phase of the project made it difficult to mobilise participants, but we elicited comprehensive information from experienced key informants. A total of 85 interviews were conducted, including 48 Process Net-Map sessions (Table 3).

**Table 3:** Study participants

Stakeholders		
	Case1	Case 2
Farmer groups (clusters)	10	–
Community facilitators	12	7
Community-based organisations	1	3
Dairy Cooperative executives	5	–
Cooperative Union executives	5	–
Representatives of County Government	2	1
Field officers	15	4
Project officers	3	2
Monitoring and Evaluation officers	1	1
Project Managers	1	1
Staff of ‘UNIQUE’ (technical capacity provider)	1	1
Staff of Vi Agroforestry	3	2
Staff of Brookside	2	–
Staff of Livelihoods Venture	2	–
Sub-total	**63**	**22**
**Total**	**85**	

### Description of the Process Net-Map Tool

The application of Process Net-Map followed three steps. Firstly, the respondents were asked to describe the entire implementation process of SACP, and at each stage, respondents identified all actors involved. The names of the actors mentioned by the respondent were written on sticky notes (so-called actor cards) by the interviewer and placed on a large flip chart sheet. Arrows were drawn between actors to indicate the steps. Each link (arrow) was numbered and the respective step of implementation corresponding to each number was described in a legend. The links were drawn with different colors to indicate the types of processes identified during the activities.

Secondly, the interviewer asked the respondents to determine how much influence each actor had on the outcome, which was defined as the adoption of SALM and dairy practices. For visualisation, the researcher stacked checker game pieces on top of each actor to build so-called influence towers for each actor on a predetermined scale from 0-8, based on respondents perceived level of influence. The height of the tower depicts the level of influence assigned to an actor by the interviewees. The influence towers were placed next to the actors, and actors with 8 checker pieces were perceived to be the most influential in ensuring the adoption and establishment of SALM and dairy practices. Those actors with no checker pieces were considered not to have any influence on the outcome. Respondents were asked to give reasons for assigning specific influence levels to a particular actor.

In the third step, the researcher used the map to ignite a discussion on the governance challenges. The respondents were asked to identify potential challenges and determine at which stage these challenges are likely to occur in the implementation process. This was followed by a discussion on how these challenges were addressed. The facilitator probed for existing digital tools and how their application can help solve some of these challenges.

Given that respondents had in-depth experience and insight in different areas of SACPs, individual maps were first compared and then combined based on discussions with stakeholders who have a broader insight into all stages of SACPs. The first two authors conducted the mapping and interviews with groups and individual respondents. All sessions and interviews were audio recorded with prior permission from the participants. Participation in field officers’ training and visits to farms, dairy cooperatives, and CBOs was used to triangulate interview results.

The individual Process Net-Maps were aggregated by the authors into a single simplified map showing consecutive steps, linkages between actors, levels of influence and challenges in implementing the SACPs. Content analysis was used to analyse in-depth interviews, FGDs, and discussions around governance challenges and mitigation strategies. Content analysis is considered a useful tool for exploratory and descriptive studies, particularly in collaborative studies where participating subjects are also stakeholders in a situation in need of change or action (Adu-Baffour et al. 2021; Simms and Erwin 2021). In addition to the qualitative findings, frequencies of how often specific challenges were mentioned, are reported.

## Results

### Implementation of Smallholder Agricultural Carbon Projects

**Fig. 2:**
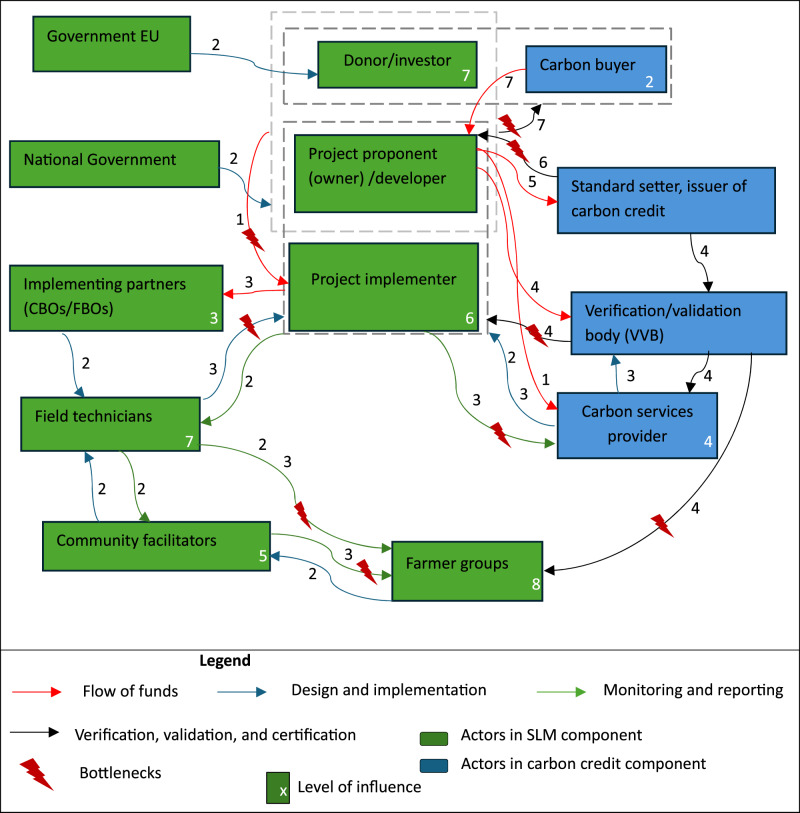
Key components and functions of a generic model of smallholder agricultural carbon project

The implementation of SACPs follows the generic structure of agricultural carbon projects as shown in the conceptual framework. Our analysis identified a few more relevant actors in each SACP component who play a role in the identified challenges. Figure 2 shows the general structure of SACPs, indicating the key actors, their roles, and how they are linked, the systematic steps in implementing SACPs (numbers on arrows), and where governance challenges arise (lightning bolts). As shown in the conceptual framework, SACPs have two main components, namely ‘SLM and carbon credits’. The SLM component consists of the actors shown in green on the left side of Fig. 2, while the carbon credit component is shown in blue on the right side of Fig. 2. The systematic steps in the implementation of SACPs in the case studies are presented in Appendix (Table 6).

### Governance Challenges of SACPs and Mechanisms Employed

This section presents the empirical findings on the governance challenges affecting the two components of the SACPs and the strategies being employed (Table 4), as respondents reported. The results of the two cases were pooled because there was no significant difference between the responses. Despite differences in funding and payment structures, the cases exhibit the same challenges and underlying reasons. It is the intensity of a few challenges that varies, rather than the type. The identified governance challenges are also highlighted in Fig. 2 in the form of lightning bolts.

**Table 4:** Major Governance challenges of SACPs and mitigation strategies

Challenges	%	Underlying reasons	Bargaining power dimension	Mitigation strategies
*SLM Component*				
Adoption of SALM and dairy practices	51	∙ gender roles and local norms ∙ nonparticipation in project design ∙ unavailability of some inputs, such as fodder grass seed ∙ small land size and prioritization of food security ∙ delay in supply of inputs (tree seedlings, fodders, etc) ∙ budgetary constraints	∙ Limited decision-making power of primary implementers (women) ∙ weak control over land and inputs reduces ability to comply with project requirements	∙ participatory project design where participating farmers are consulted regarding project activities ∙ connect input suppliers with farmers at no cost
Participation and awareness	45	∙ lack of transparency ∙ poor expectation management ∙ limited participatory decision-making ∙ gender roles (women participate but no land ownership and decision-making power)	∙ Participation without bargaining power; women supply labor but lack voice and control over project decisions	∙ participatory project design ∙ more efforts from project managers to create awareness and capacity (trainings, information materials, and presentations)
Technical capacity	42	∙ knowledge gap on carbon credit ∙ local elite ∙ low sensitization ∙ inadequate number of field officers	∙ Asymmetric access to information and technical knowledge reinforces elite bargaining advantage	∙ local capacity development ∙ information sharing platforms ∙ participatory decision-making mechanism ∙ intensive training on voluntary carbon market
Accountability of major decision makers*	42	∙ low local capacity ∙ power imbalances ∙ elite capture ∙ little knowledge on voluntary carbon credits ∙ incomplete contracts	∙ Weak downward accountability due to unequal bargaining positions and inability to contest decisions	∙ participatory decision-making mechanism ∙ local capacity development ∙ training on how voluntary carbon market works
Risk on weaker actors	16	∙ power imbalances ∙ top-down project design	∙ Risk shifted to actors with weakest bargaining power and fewest outside options	∙ participatory decision ∙ information sharing platforms ∙ mechanisms for expectation management
*Carbon credit component*				
Transparency (mainly about carbon credit)*	81	∙ power imbalances ∙ top-down design ∙ low local technical capacity ∙ limited knowledge regarding carbon credit	∙ Upstream control over carbon information and certification creates opacity for downstream actors	∙ capacity building on voluntary carbon market ∙ participatory decision making ∙ bottom-up decision process
Extension vs data collection*	72	∙ low incentives ∙ costs of extension ∙ monitoring demands ∙ power imbalances ∙ complex certification requirements ∙ non-participatory design	∙ Monitoring priorities set by powerful principals crowd out support functions, increasing agent burden without compensating authority	∙ farmer incentives required ∙ local capacity building ∙ use of simple digital tools by farmers and CFs for data collection and analysis

Note: % = percentage of responses; * = the challenges that were more pronounced in Case 1

#### SLM Component

**Fig. 3:**
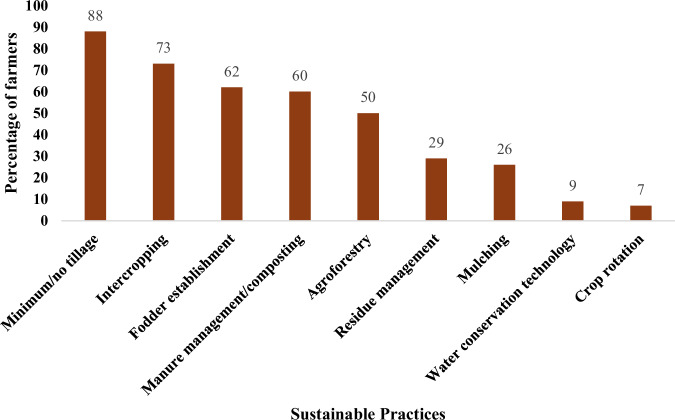
Adoption rate of promoted sustainable practices

The SLM component faced adoption challenges, particularly among women farmers who make up over 70% of project participants but lack land ownership and decision-making power due to gender norms. Figure 3 summarises the adoption of the promoted practices. As men typically decide which SALM practices to adopt, this constrained implementation and farmland tracking. The following were illustrated by a cooperative leader (Case 1) and a community facilitator (Case 2), respectively.

*“…so you will find the lady is the one in the project and not the husband; and so, when they [field officers] came in and wanted to measure the land; the husband says no; this is my land; what do you want to do with my land: why do you measure my land; do you want to sell my land”*.

*“Some of my group members couldn’t plant the tree seedlings because the Muzee [man] didn’t agree. Some husbands also harvested the trees prematurely without the women’s consent”*.

In six of the ten FGDs, respondents reported that cultural norms encapsulated by the phrase ‘Ng’ombe ni Baaba’ (cattle belong to men) assign ownership of cattle and control of milk income to men, although women perform most daily dairy tasks. This results in unequal distribution of benefits within households, whereby the male household head is given the morning milk, which is then sold to cooperatives under accounts largely registered in men’s names. Meanwhile, the evening milk is used for household consumption. Only the surplus is accessible to women. This is sometimes sold to neighbors. Adoption of SALM practices was further constrained by delayed or unavailable inputs, budget limitations, small plot sizes, and food security priorities that limit space for practices such as agroforestry. One field officer (Case 1) explained:

*“Food security is very important in this project, so adoption of these practices is a gradual process since farmers have small plots and need to also grow food crops to feed their families. Since about 50% to 70% of the farmers are dairy producers, it will take time for the farmer to understand that milk sales are more profitable than the maize that they grow; the point is with time farmers will understand and produce more fodder, get more milk, and use the money to buy food stuffs instead”*.

To address the issue of gender norms, project implementers used a participatory household approach (Household Road Map) to train households on sharing responsibilities within the household. In four out of 10 FGDs participants described that the training helped men to support their wives in feeding the cows and planting fodder, although some households did not participate.

Furthermore, respondents attributed low participation in voluntary carbon markets to limited awareness, top-down project design, and information asymmetry. In Case 2, top-down decision-making shifted risks onto farmers. Five out of seven community facilitators reported significant crop losses due to the fall armyworm in 2018. These losses were attributed to pesticide restrictions imposed as part of the project’s compliance regulations. Moreover, due to budget constraints, one field officer was responsible for supervising over 80 dispersed farmer groups, which limited access to training. Farmer group leaders made decisions without accountability, partly due to low technical capacity. Surprisingly, none of the interviewed farmers had copies of project contracts, although they were all asked to sign a contract. The following statement by a group leader who is also a community facilitator (Case 2), when asked why farmers do not have a copy of the project contract, reflects these findings:

*“We don’t know that we can ask for a copy of the contract document and if some people know, we don’t have the boldness to ask ViA”*.

#### Carbon Credit Component

The carbon credit component faced two key challenges: limited transparency and trade-offs between intensive data collection and extension service delivery. These issues stemmed from a top-down approach, power imbalances, limited local capacity in voluntary carbon markets, and asymmetric information among stakeholders. One field officer (Case 1) explained:

*“The debate on whether smallholders should know about carbon credits is still open, … and I will not tell farmers about it if I am not instructed to do so by management”*.

In addition, complex and extensive data requirements driven by investment risk, high transaction costs, limited incentives, and low levels of farmer education reduced the availability and quality of training for participating farmers. One staff member of the project proponents stated:

*“…you know the major aim of this carbon project is to improve the livelihoods of smallholder farmers and this is why our carbon is considered as 5-star, but I am worried because now the attention has been shifted to intense data collection”*.

To address the issue of intensive data collection, a carbon technical capacity provider (UNIQUE) developed a mobile app for digital data collection. The field officers indicated that the tool can potentially improve data accuracy, but it is time demanding (mentioned by 13 out of 15 field officers).

### Role of Digital Tools in SACPs

This study found that digital tools are only being used in the SLM component of SACPs, suggesting untapped potential in the carbon credit component. A summary of the findings is presented in Table 5. The interviews revealed that community facilitators use SMS to send data on food production, fertiliser, herd size and trees to the project implementer’s server. Field officers track farms involved in carbon projects with GPS. Although virtual communication platforms are used for project updates and regular communication between top-level actors, there is a lack of digital systems that share project information among all stakeholders. Our discussions also revealed the potential of digital tools to reduce transaction costs. Further detailed discussions revealed respondents’ perspectives on the implications of the digital tools in use.

**Table 5:** Role of digital tools

Project function	Digital tools in use	P-A implications	Implications for bargaining power
Data collection and reporting	∙ Mobile applications used by field officers ∙ SMS-based reporting by community facilitators ∙ Central database for data storage	∙ Reduces information asymmetry for upstream principals ∙ Strengthens monitoring of agents (farmers, CFs)	∙ Increases the compliance burden on local actors without improving their bargaining power or access to information
Farm tracking and verification	∙ GPS tracking of participating farms	∙ Enhances oversight and traceability for principals ∙ Supports verification requirements	∙ Reinforces upward accountability ∙ Farmers have limited control over how data are used
Stakeholder engagement	∙ Virtual communication platforms among top-level actors (project developers, technical providers)	∙ Facilitates coordination between principals and intermediaries	∙ Excludes farmers and local actors ∙ Reinforces information and decision-making asymmetries

### Benefits and Beneficiaries in SACPs

This study shows that the identification of beneficiaries and benefits depends on the eligibility criteria for participation, the source of project funding, the type of benefit (monetary or non-monetary), the performance of the group, and the overall objective of the project. Case 1 beneficiaries include farmer groups that receive non-monetary benefits (training on sustainable practices, seedlings, and fodder planting material), and dairy cooperatives that receive business management training, as well as international investors that receive carbon credits to offset their emissions. Beneficiaries in Case 2 include farmer groups, who receive both monetary and non-monetary benefits, and the donor organisations, which also are buyers of carbon credits. It is unclear from our study what proportion of the funds is allocated to each group of actors. Field officers indicated that the opportunity costs of land use change and the costs incurred by each actor group were not considered in the implementation and benefit sharing processes (13 out of 19). The carbon benefit is derived from the sale of carbon credits generated by smallholders. In both cases, there is an information gap on the benefit sharing mechanism, as this was determined solely by top-level actors, without the participation of farmers. For example, Fig. 2 shows that farmer groups are the most powerful actors (influence level 8) in terms of adopting sustainable practices, but their voice was not considered in benefit sharing.

The study also identified trade-offs in benefit-sharing arrangements. For example, group benefit sharing can potentially reduce transaction costs but can result in free-riding members receiving benefits. Furthermore, performance-based benefit sharing can encourage continued adoption, but it requires robust strategies to monitor group performance, which increases monitoring costs and affects the benefits farmers receive.

## Discussion and Recommendations

The present study demonstrates that the governance challenges identified in SACPs stem from asymmetries of bargaining power within the chain of P-A relationships. In line with the conceptual framework outlined in Section Two, viewing these challenges through the two-component framework highlights how the carbon credit component reshapes and intensifies existing SLM governance challenges. The digital tools identified also demonstrate their limited application to the SLM component only. This section discusses key governance challenges in the context of P-A relations, bargaining power, and the role of digital tools.

### Key Challenges of the ‘SLM Component’

#### Participation with Limited Power and Incomplete Agency

The findings showed that adoption problems arise at the design and implementation stages, and at the monitoring and reporting stages of SACPs (Fig. 2). In both cases, a top-down design approach was used. Limited participation and awareness stem from top-down project design, where principals retain decision-making authority and agents are informed primarily at the implementation stage. Smallholders’ limited knowledge regarding carbon sequestration, the generation of carbon credits, and how the carbon market works contributes to information asymmetries that weaken their bargaining power (Awazi et al. 2025). The lack of farmer participation in project design suggests that farmers’ socioeconomic and environmental conditions may not have been considered in selecting project activities, which may negatively affect adoption and yield, thus raising concerns about sustainability, long-term benefits and contributions to food security of the project.

In terms of gender, the findings show that the socio-cultural context is extremely relevant to the effectiveness of the project but has not been sufficiently considered. Women are the project’s core participants and labor providers, but they have limited decision-making power over project design, land use, and benefit sharing due to existing gender norms, which reflect constraints on their agency. This creates a mismatch between labor provision and decision-making power, which is consistent with the gendered P-A problem at the intra-household level (Agarwal, 1997). Women are expected to comply with project requirements, yet they have limited bargaining power over key resources, including land and livestock. This constraint is further exacerbated by small land sizes and delayed input supply, which reduces the potential for adopting SALM and dairy practices, even when incentives are nominally aligned. From a P-A perspective, while the contracts are formally incentive-compatible, they are substantively problematic given the agent’s constrained control over key resources.

Moreover, the high rate of women participation in SACPs does not result in control over benefits. Their bargaining power, which could be strengthened through collective action, is limited by elite capture and project incentive structures that offer little motivation for collective negotiation. While much of the labor required for SALM adoption and carbon monitoring is supplied by women, the co-benefits such as milk revenue are predominantly accrued by men due to gendered asset ownership and market access. This suggests that participation without bargaining power does not alter the distribution of benefits. This is consistent with the findings of other studies on PES or land-based projects. For example, Kariuki et al. (2018) found that land-based conservation schemes mostly benefit men. Women make up over 70% of the participating farmers, and the level of farmer influence suggests that the power to adopt or establish SALM and dairy practices rests with them. Women have been found to be more efficient and competent than men in implementing practices (Shames et al. 2016).

These gender issues also suggest that the sustainability of SACPs depends largely on the ability of women to continue to adopt most SALM and dairy practices. Strengthening dairy cooperatives can increase their bargaining power and make them competitive in the milk market, while improving men’s participation in SACPs as most accounts are in men’s names. To address this inequality, capacity building of farmer groups and participatory project design are crucial. It is recommended that project developers should consider gender and less labor-intensive practices when developing appropriate practices to increase participation. Given the complex nature of project design, which involves different actors who have not previously worked together toward a common goal, it is necessary to build the decision-making capacity of all actors to reduce power-based inequalities.

#### Accountability Gaps and Power Asymmetries

Lack of accountability of major decision-makers and the risk exposure of weaker actors are key factors in shaping governance outcomes in SACPs, highlighting the central role of bargaining power. Incomplete contracts, elite capture, and limited capacity restrict farmers’ and community-based organizations’ ability to hold upstream actors accountable. Within the layered P-A relationships, authority, information, and contractual control tend to be concentrated at the top, while implementation and performance risks tend to accumulate at the bottom, where bargaining power is weakest. Consequently, local actors lack the institutional capacity to contest decisions, and project risks such as failure to meet adoption or performance targets are effectively transferred downward. This is consistent with the proposition that bargaining power determines how contractual gaps are filled under conditions of incomplete contracting (Hambloch et al. 2026).

The capital-intensive nature of SACPs, particularly during the initial implementation and monitoring phases, exacerbates these dynamics. The high transaction costs associated with contract negotiation, monitoring, and establishment (Shames et al. 2016) highlight the importance of securing multiple funding sources, particularly for participatory project designs, where transaction costs are higher. Therefore, schemes that provide farmers with direct cash payments from carbon revenues alongside extension services are critical to sustaining participation and adoption. Such payments can alleviate financial constraints, enabling farmers to invest in resources and practices that would otherwise be out of their reach (von Braun et al. 2023; Lal et al. 2018). However, this study finds that carbon payments are often minimal, with a significant proportion of revenues being absorbed by monitoring, verification, and validation costs. This is consistent with the findings of Cavanagh et al. (2021). This finding is also consistent with findings from the N’hambita Community Forest Carbon Project, where transaction costs and intermediaries captured most carbon revenues (Jindal et al. 2012). Other studies attribute low payments to low carbon prices and the prioritization of productivity-related co-benefits (Schilling et al. 2023; Lee, 2017).

Limited financial resources can undermine the quality of data collection and reporting, which could compromise the credibility of carbon claims. Low education levels among farmers and weak incentives for community facilitators reduce engagement in monitoring activities, exacerbating P-A problems. Digital platforms, such as e-extension services and simple handheld tools for measuring soil organic carbon, offer the potential to reduce advisory and verification costs while strengthening local capacity (Daum et al. 2022; Shames et al. 2013). However, their effectiveness depends on whether they are embedded in governance arrangements that enhance local bargaining power rather than merely lowering compliance costs for upstream actors.

### Beyond SLM: Governance Trade-offs of Incorporating Carbon Credits

The prevalence of transparency concerns in SACPs reflects their hierarchical governance structure, in which carbon buyers and certification standards act as powerful upstream principals. Control over technical knowledge, MRV methodologies and certification processes concentrates bargaining power at the top of the project chain, leaving farmers and other field-level stakeholders with limited visibility of carbon accounting, pricing, and revenue flows. This lack of transparency undermines accountability and trust, as key decision-makers face limited downward scrutiny. Disputes over carbon payments commonly arise from poor transparency, insignificant payments, and poorly defined benefit-sharing mechanisms (Shames et al. 2016). Although the importance of equitable carbon revenue sharing has been widely recognized (Foster and Neufeldt 2014), the absence of standardized mechanisms across certification systems remains a persistent challenge (World Bank Group, 2019).

From a P-A perspective, the carbon credit component aims to minimize information asymmetries between buyers and project developers by imposing rigorous monitoring and verification requirements. However, these mechanisms create new agency problems further down the chain, as field managers and farmers are burdened with increased reporting and compliance responsibilities without the corresponding authority, incentives or access to information. Consequently, responsibilities and risks are shifted downwards while control over decisions and financial flows remains concentrated upwards. These dynamics are further reinforced by intra-household bargaining structures, whereby women often undertake monitoring and adoption labor while having limited control over co-benefits. As a result, existing gender inequalities may be reproduced by SACPs despite high levels of women participation.

The tension between extension services and data collection illustrates how monitoring demands can crowd out support functions, thereby undermining SLM practices, which are ultimately crucial for carbon performance. When projects are perceived as prioritising carbon targets over livelihood benefits, farmer trust and motivation erode, which weakens long-term participation (Oldfield et al. 2022). Proposed mitigation strategies, including local capacity building, simple digital tools, and improved farmer incentives, aim to reduce information asymmetries without disproportionately increasing agent burdens. However, technical solutions alone are insufficient if underlying power imbalances persist.

In recent years, Verra and the Integrity Council for the Voluntary Carbon Market (ICVCM) have sought to enhance the credibility of the carbon market. Verra revised its VCS Program, releasing VCS Version 5.0 in December 2025. This version emphasizes inclusive project design, transparency, stakeholder engagement, safeguards, clearer rights and claims, stronger grievance mechanisms, and more credible benefit-sharing where rights-holders are affected. The ICVCM’s Core Carbon Principles (CCPs) complement these reforms by setting market-wide governance, transparency and safeguard benchmarks, helping buyers identify higher-integrity credits.

However, it remains uncertain how far core challenges can be reduced in practice: entrenched gender norms and intra-household bargaining may still shape who bears labor input, who takes decisions and who controls benefits. Likewise, the tension between extension support and data collection may persist as standards become more rigorous, MRV more complex and transaction costs rise. Because projects remain fundamentally compliance- and audit-driven and typically financed through upstream buyers or investors, basic power imbalances and top-down control over methodologies, certification choices and revenue timing are likely to endure even as formal safeguards improve. The sheer scale of these projects will continue to challenge participatory design and transparency. Our research, therefore, points to prioritizing low-transaction-cost measures that protect trust at scale, with clear communication e.g. a “carbon factsheet”, clearly defining extension times and activities, not necessarily tied to monitoring activities but using the same institutional structure. Projects may additionally pursue CCB certification to strengthen claims on community and biodiversity outcomes, but only where the added requirements are matched by market access or pricing rather than further pressure on project economics.

### Digital Tools and Governance in SACPs

The use of digital tools in the SACPs is primarily limited to the SLM component to reduce information asymmetries along the P-A chain and lower transaction costs for project implementers. These tools strengthen upward accountability by improving oversight of compliance with project requirements at farm level. However, their potential to address broader governance challenges is limited by the lack of integrated digital systems that allow information sharing across all stakeholders. Particularly, digital tools have not been utilised for project planning, carbon monitoring or benefit distribution, thereby neglecting critical facets of the carbon credit component overseen through centralised systems.

From a bargaining power perspective, the selective deployment of digital tools reinforces existing power asymmetries rather than transforming them. Although farmers and community facilitators supply data through digital systems, control over data analysis, carbon accounting, and financial flows remains concentrated among project developers and technical providers. However, sharing experiences and data among smallholders can influence learning and best practices (Daum et al. 2022). The absence of digital payment and benefit distribution tools undermines farmers’ ability to claim or negotiate benefits, while maintaining intermediary control over revenues. The untapped potential of digital MRV technologies suggests not only technical constraints, but also institutional choices that preserve expert authority and hierarchical governance. Without investment in local capacity and inclusive digital systems, digital tools risk exacerbating monitoring burdens, particularly for women, without enhancing their bargaining and decision-making power.

### Limitations and Further Research

Carbon projects and schemes are a complex and sensitive topic due to the range of actors involved and the many governance challenges. Therefore, it was essential to build trust with interviewees, conduct longer interviews, and carry out an in-depth study. A key limitation of this study is the inability to engage with independent carbon standard-setting organizations, such as Verra and Gold Standard. Data collection in Case 2 was limited but key persons interviewed in the project had sufficient knowledge of the project and provided comprehensive information. Another limitation is that socio-economic aspects apart from gender was not considered. Although this was not mentioned in the interviews, challenges might exacerbate for participants with lower education levels, less land, and income.

While the current study used a qualitative approach to identify the underlying reasons for governance challenges, a survey could be used to identify the extent of the challenges. The challenges identified in this study may also arise in other complex and PES related programs and projects, including in the revised Verra methodologies. Therefore, the strategies used to address the identified governance challenges can guide current and future project managers, project developers, and policymakers in designing appropriate strategies for the implementation of not only SACPs but also other related complex projects involving smallholder farmers, especially in developing countries.

## Concluding Remarks

This paper analyzed the governance challenges of SACPs from the perspectives of P-A theory and bargaining power theory, as well as considering the role of digital tools. The findings reveal that these challenges originate from the dual structure of SACPs and how responsibilities, risks, and benefits are allocated. The carbon credit component introduces layered P-A relationships characterized by strict monitoring and external accountability, which exacerbates information asymmetries and shifts performance risks to actors with limited bargaining power. Gender norms further shape these dynamics. Although women play a key role, entrenched intra-household power relations limit their control over assets and benefits. Consequently, increased women’s participation does not improve bargaining positions, thereby reinforcing gender inequities within SACPs, which can negatively affect carbon performance and lead to costly dropouts and communication challenges. The study also highlights hierarchical power structures in terms of where the money goes, with extension vs. monitoring being a key example.

Digital tools have the potential to mitigate some of the problems associated with P-A relationships by reducing information asymmetries, lowering transaction costs and strengthening accountability. Currently, however, they are mostly used for data collection, which reinforces monitoring rather than empowering local actors. However, expanding their use to support shared project planning, transparent benefit distribution and feedback on carbon performance could enhance farmers’ bargaining power and rebalance incentives. Participatory governance arrangements and inclusive public digital infrastructure are essential to achieving these outcomes, ensuring more equitable and effective carbon governance rather than reproducing existing power asymmetries.

## Data Availability

The data used in this paper are confidential.

## Appendix

**Table 6:** Major steps in SACPs implementation

Steps	Description	
	Case 1 (Livelihoods Mt. Elgon)	Case 2 (Kenya Agricultural Carbon Project)
**1. Release of funds**	**Livelihoods fund(investor):** released funds to Unique Forestry (Carbon technical capacity provider) and Vi Agroforestry (project implementors) in addition to a grant from a co-investor (Brookside dairy limited), in accordance with EU and national governments guidelines.	**World Bank (donor):** released fund to Vi Agroforestry (ViA) (the project developer/ implementor) and Unique Forestry (Carbon technical capacity provider).
**2. Design and Implementation**	**ViA and field technicians:** organized stakeholder engagement, recruited, formed, and registered farmer groups and clusters, conducted baseline survey, trained farmer groups on SALM, dairy management, procured and distributed fodder materials (starter seeds) to farmer groups, and conducted baseline survey. **Farmer groups:** signed farmer commitment forms and elected community facilitators (CFs).	**ViA and field technicians:** mapped actors and project areas, stakeholder engagement to develop working modalities, community entry, and conducted baseline survey. Recruited farmer groups, tracked farms, trained farmers, and rolled out activities with farmers. Procured tree seedlings and distributed to farmers and formed clusters. **Farmer groups:** signed farmer commitment forms and rolled out activities, elected CF.
**3. Monitoring and reporting**	**ViA:** contracted implementing partners (CBO) to supervise field technicians, submits monitoring report to UNIQUE, **CFs and Field technicians:** internal verification and submission of group summary data to the database via sms **Farmer groups:** collected self-assessment data on SALM practices, farm output, including daily milk yield, and number of trees **Carbon technical capacity providers:** developed database, calculates sequestered/ reduced carbon	**Farmer groups:** filled annual self-assessment data on number of trees, farm output, SALM practices, **CFs:** submitted self-assessed data (group summary) to a database **ViA and CBOs:** internal verification of data and submission of monitoring report to Unique **UNIQUE:** developed database and calculates sequestered carbon
**4. Validation and verification (auditing)**	**Project developer:** contracts TÜV NORD (verifier) to conduct validation and verification. **UNIQUE:** submits monitoring report to the verifier **TÜV NORD:** organizes stakeholder engagement and visited sampled farmers and farms for auditing. Provides validation report and certificate to project developer	**Project implementer:** contracted TÜV NORD (verifier) to conduct validation and verification **UNIQUE:** submits monitoring report to the verifier **TÜV NORD:** organizes stakeholder engagement and visited sampled farmers and farms for auditing. Provides validation report and certificate to ViA
**5. Registering with standard setters**	**Project proponent/developer (Livelihoods):** registered the project at the registry of standard setters (Verra and Gold Standard), provided project documents including validation and verification reports and certificate obtained from verifier	**Project developer/implementer (ViA):** Registered the project at Verra registry, provided project documents including validation and verification reports and certificate obtained from verifier
**6. Issuance of carbon certificate**	**Certifier (Verra and Gold Standard):** issues carbon certificate (carbon credit) to project proponent after successful assessment of project documents	**Standard setters (Verra):** issues carbon certificate (carbon credit) to ViA after successful assessment of project documents
**7. Carbon credit marketing**	**Project proponent:** distributes carbon credit proportionately to the investors for the project period and ViA will identify buyers, negotiate price, and sell carbon credit afterwards	**ViA: sell first credit to the donor and later i**dentifies credit buyers, negotiates price, and sells carbon credit and distributes carbon revenue to farmer groups based on performance

Note: Steps 3-7 are repeated every 2–5 years of project life

## Footnotes

1 SACPs is used in this study to represent smallholder agricultural carbon projects of the AFOLU project categories: (1. Afforestation, Reforestation and Revegetation (ARR); 2. Improved Forest Management (IFM) 3. Reduced Emission from Deforestation and forest Degradation (REDD) 4. Avoided Conversion of Grassland and Shrubland (ACoGS) 5. Agricultural Land Management (ALM) and 6. Wetland Restoration and Conservation (WRC).

2 Verra’s SALM methodology has been inactivated in 2023. Existing projects can still follow the SALM methodology, new projects have to use similar methodologies. Source: Verra, 2023 (https://verra.org/verra-announces-planned-inactivation-of-sustainable-agricultural-land-management-methodology-vm0017/#:~:text=Verra%20will%20inactivate%20VCS%20methodology%20VM0017%20Adoption%20of,the%20VCS%20Methodology%20Development%20and%20Review%20Process%2C%20v4.2).
